# MethPed: an R package for the identification of pediatric brain tumor subtypes

**DOI:** 10.1186/s12859-016-1144-0

**Published:** 2016-07-02

**Authors:** Mohammad Tanvir Ahamed, Anna Danielsson, Szilárd Nemes, Helena Carén

**Affiliations:** Sahlgrenska Cancer Center, Department of Pathology, Institute of Biomedicine, Sahlgrenska Academy, University of Gothenburg, PO Box 425, SE-405 30 Gothenburg, Sweden; Sahlgrenska Cancer Center, Department of Oncology, Institute of Clinical Sciences, Sahlgrenska Academy, University of Gothenburg, PO Box 425, SE-405 30 Gothenburg, Sweden; Swedish Hip Arthroplasty Register, Centre of Registers Västra Götaland, Gothenburg, PO Box 425, SE-405 30 Gothenburg, Sweden

**Keywords:** DNA methylation, 450K, Random forest, R package, Glioblastoma, Medulloblastoma, Ependymoma, Classifier (classification tool), Astrocytoma, MethPed

## Abstract

**Background:**

DNA methylation profiling of pediatric brain tumors offers a new way of diagnosing and subgrouping these tumors which improves current clinical diagnostics based on histopathology. We have therefore developed the MethPed classifier, which is a multiclass random forest algorithm, based on DNA methylation profiles from many subgroups of pediatric brain tumors.

**Results:**

We developed an R package that implements the MethPed classifier, making it easily available and accessible. The package can be used for estimating the probability that an unknown sample belongs to each of nine pediatric brain tumor diagnoses/subgroups.

**Conclusions:**

The MethPed R package efficiently classifies pediatric brain tumors using the developed MethPed classifier. MethPed is available via Bioconductor: http://bioconductor.org/packages/MethPed/

## Background

Carcinogenesis involves changes in gene expression that results in tumor specific gene and protein signatures. Such signatures have been used to classify different subtypes of cancers. Gene expression is partly regulated by the methylation state of CpG islands. Cancer tissue is characterized by an increased variability in DNA methylation patterns. DNA methylation profiling has been reported as a robust method to classify and subgroup tumors of different origin [1]. For most pediatric brain tumor diagnoses, methylation profiling can divide the tumors into clinically relevant subgroups reflecting the diverse biology of the different subtypes which further highlights the need for specific therapeutic strategies to target different subgroups. With the increased knowledge about specific brain tumor subgroups and the development of targeted therapy for different entities, it is essential to quickly and accurately determine the correct diagnosis for pediatric brain tumor patients. The most popular and commonly used platform for genome-wide methylation profiling is the Illumina Infinium Human Methylation 450 BeadChip arrays. These arrays profile ~485,000 CpG sites and have been used by the Cancer Genome Atlas Project (TCGA) and in numerous studies of pediatric brain tumors. A correct diagnosis is vital for determining the appropriate treatment protocol for a specific patient, to select the right patients for clinical trials investigating novel therapy for specific diagnoses and subgroups and for basic researchers to be able to draw correct conclusions from experiments. We therefore developed the MethPed classifier [2], which is a multiclass random forest algorithm [3], based on DNA methylation profiles from many subgroups of pediatric brain tumors. We have now developed an R package that uses this method, making it easily available and accessible.

## Implementation

**Fig. 1:**
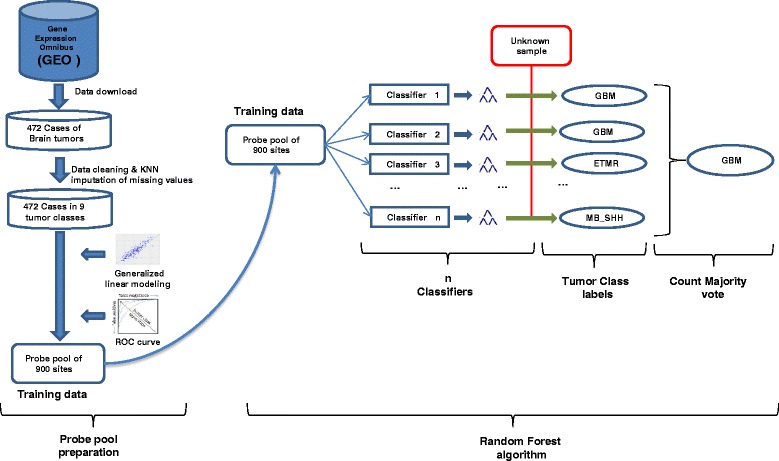
Implementation and workflow of the MethPed classifier and package

The MethPed classifier was developed using the Random Forest (RF) algorithm [3] for robust classification of unknown brain tumor samples into subtypes as described in Danielsson et al. [2]. Briefly, the RF algorithm was applied on beta values which are the estimate of methylation levels (between 0 and 1 with 0 being unmethylated and 1 fully methylated) using the ratio of intensities between methylated and unmethylated alleles generated by the Illumina Infinium HumanMethylation 450 BeadChip array. A training probe pool of 900 methylation sites that showed the highest predictive power (AUC values) in a large number of regression analyses was selected from 472 clinically diagnosed brain tumor cases available on GEO after necessary data cleaning and KNN imputation of missing values. The RF algorithm was then applied to classify unknown samples based on the selected training probe pool. See Fig. 1 for a summary of the workflow for the MethPed classifier and R package.

The MethPed classifier can be accessed through the ‘MethPed’ package that can be downloaded from Bioconductor, a repository for bioinformatics related applications. The ‘MethPed’ package includes the MethPed classifier and an example data set of two tumors. The example data can be read into the R computing environment with the help of the data function after installing the package.

Data for running MethPed is generated by the Illumina Infinium HumanMethylation 450 BeadChip arrays. Beta values for two samples (Tumor A and Tumor B) are provided with the ‘MethPed’ package as an example [2]. This data has no missing values. If missing values exist in a data set, the impute package can be used for missing value imputation, according to the MethPed vignette in Bioconductor.

## Results and discussion

**Fig. 2:**
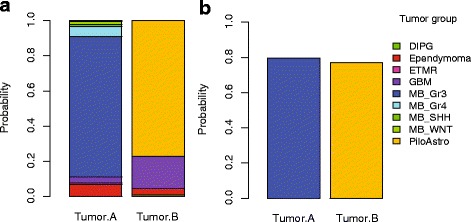
Bar plots on the diagnosis prediction of the two test samples. **a** Classification probability of a sample belonging to each of the pediatric brain tumor diagnoses currently included in MethPed and (**b**) Maximum classification probability of a specific tumor group for each sample

The MethPed analysis starts with loading the data, checking for missing values in the data file and thereafter runs through the classification. Error rate of the prediction is estimated and the probability that a sample belongs to one of nine tumor diagnoses/subgroups is given. In the current version of MethPed the following groups are included; glioblastoma (GBM), pilocytic astrocytoma, medulloblastoma (Wnt, Shh, group 3 and group 4), diffuse intrinsic pontine glioma (DIPG), ependymoma and embryonal tumor with multilayered rosettes (ETMR). These include the most common diagnoses and subgroups but not all. The robustness of a classifier is highly dependent on the accuracy of the training data and therefore we choose not to build in groups with limited data avaliable. With the MethPed classifier, the probability that a tumor sample belongs to a specific tumor group is presented (Fig. 2a), or if preferred, the group with the maximum probability leaving zero to the other tumor groups by supplying an extra parameter ‘prob = FALSE’ in the classifier (Fig. 2b). It should be note that for tumors belonging to diagnoses that are not included in the MethPed classification, these will be classified as inconclusive (with low probabilities of belonging to any of the groups) or to the most similar tumor form that is present in the classifier. For more information, see Danielsson et al. [2].

The conditional probability matrix of the classification from the MethPed output can be observed by the ‘summary’ command in R. For visualization of the prediction, bar plots can be generated by using the ‘plot’ command (Fig. 2). If missing probes from the sample compared with the training data included with the package exist, these can be observed by the ‘probeMis’ command.

MethPed is currently the only publically available tool for classification of pediatric brain tumors. The use of methylation profiling for classification of these tumors adds new and important knowledge in the clinical setting for choosing the optimal care and treatment of these patients and will therefore likely complement histopathological diagnoses in the near future [1, 2].

## Conclusions

The MethPed R package can be used to efficiently classify pediatric brain tumors using DNA methylation profiles generated by the Illumina 450 K methylation arrays.

## Abbreviations

DIPG, diffuse intrinsic pontine glioma; ETMR, embryonal tumor with multilayered rosettes; GBM, glioblastoma; TCGA, the Cancer Genome Atlas Project.
